# Getting in (and out of) the loop: regulating higher order telomere structures

**DOI:** 10.3389/fonc.2012.00180

**Published:** 2012-11-30

**Authors:** Sarah Luke-Glaser, Heiko Poschke, Brian Luke

**Affiliations:** Zentrum für Molekulare Biologie der Universität Heidelberg (ZMBH), DKFZ-ZMBH AllianzHeidelberg, Germany

**Keywords:** t-loop, telomere, RTEL1, end protection, cancer, Mph1

## Abstract

The DNA at the ends of linear chromosomes (the telomere) folds back onto itself and forms an intramolecular lariat-like structure. Although the telomere loop has been implicated in the protection of chromosome ends from nuclease-mediated resection and unscheduled DNA repair activities, it potentially poses an obstacle to the DNA replication machinery during S-phase. Therefore, the coordinated regulation of telomere loop formation, maintenance, and resolution is required in order to establish a balance between protecting the chromosome ends and promoting their duplication prior to cell division. Until recently, the only factor known to influence telomere looping in human cells was TRF2, a component of the shelterin complex. Recent work in yeast and mouse cells has uncovered additional regulatory factors that affect the loop structure at telomeres. In the following “perspective” we outline what is known about telomere looping and highlight the latest results regarding the regulation of this chromosome end structure. We speculate about how the manipulation of the telomere loop may have therapeutic implications in terms of diseases associated with telomere dysfunction and uncontrolled proliferation.

## Introduction

Telomeres protect the ends of chromosomes from being recognized as DNA double-strand breaks (DSBs) and thereby prevent the faulty repair of chromosome ends (de Lange, 2009). With the evolution of linear genomes, cells were faced with the immediate challenge of sequestering the exposed chromosome ends away from DNA repair activities such as non-homologous end joining (NHEJ) and homology directed repair (HDR). Many present-day eukaryotes have developed several lines of defense devoted to protecting telomeres from deleterious repair. Of these, the best characterized are the telomere-associated proteinaceous complexes such as shelterin, CST (Cdc13 (Ctc1)/Stn1/Ten1) and the Ku70/80 heterodimer, which work in concert to ensure that telomeres avoid being recognized as DSBs (Giraud-Panis et al., 2010). Despite these measures, telomeric ends are nonetheless subject to “controlled” exonuclease resection, which accounts for the 3′ single stranded (ss) DNA overhang that is detectable at the ends of many eukaryotic chromosome ends. As with other ssDNA substrates, the telomeric overhang is, in theory, a highly recombinogenic intermediate that can potentially engage in illegitimate recombination events with interstitial telomere repeats, resulting in deleterious chromosome rearrangements. The exposed DNA end is also at risk of being further processed by nuclease activities, which through the generation of ssDNA may activate a potent DNA damage response (DDR) and drive the cell into irreversible cellular senescence. To prevent the telomeres from either recombining or being exposed to nucleases, the telomeric 3′ overhang gets tucked away by forming a lasso-like fold-back structure, whereby the overhang forms a displacement loop (D-loop) within the telomeric tract (Griffith et al., 1999). Therefore the end gets protected, but the loop structure does not bypass the need for ss telomeric binding proteins, as they would in theory being required to coat the displaced strand of the D-loop (see Figure 2 for illustration). In mammals, the telomere fold-back structure is referred to as the t-loop, for telomeric loop. In terms of the evolution of linear chromosomes, it has been proposed that the t-loop structure may represent the most primitive means of protecting a telomere and likely arose before the protein-based telomere capping complexes (de Lange, 2004). Fold-back structures have been detected at telomeric DNA stemming from various organisms, including: *H. sapiens* (Griffith et al., 1999), *C. elegans* (Raices et al., 2008), *S. pombe* (Tomaska et al., 2004), *S. cerevisiae* (Strahl-Bolsinger et al., 1997), *P. sativum* (Cesare et al., 2003) and *T. brucei* (Munoz-Jordan et al., 2001). Although the most parsimonious model for the t-loop would be that it is providing an additional level of end-protection in conjunction with the capping complexes, the evidence to date remains correlative. The lack of understanding regarding t-loop function stems from the fact that they have been extremely difficult to detect *in vivo* in a reliable and quantitative manner, and as a result, little has been reported regarding their molecular dependencies in terms of establishment and/or maintenance. Details pertaining to: *in vivo* t-loop regulation through the cell cycle, during telomerase-mediated elongation, or at dysfunctional telomeres remains elusive. In spite of the *in vivo* shortcomings related to t-loop detection there have been many intriguing models and speculations focused on how t-loops may be regulated during the above-mentioned processes. For example, it has been proposed that such a structure has to be resolved in S-phase to allow passage of the replication fork (Vannier et al., 2012). It is also perceivable that telomerase, the reverse transcriptase that elongates telomeres, would require an “open” structure in order to facilitate interaction with the overhang before the elongation reaction ensues (de Lange, 2004). Finally, although in general HDR is suppressed at telomeres, there are pathological situations where it replaces telomerase as the mode of telomere elongation. Indeed, alternative lengthening of telomeres (ALT) tumor cells maintain their telomeres via recombination, and it is likely that t-loops have to be opened to engage in HDR with either t-circles or other telomeres (Tomaska et al., 2009). Another very plausible possibility, however, would be that the invading 3′ overhang of the t-loop gets extended within the loop itself. Such an intrachromosomal HDR event would not necessarily require loop opening (Tomaska et al., 2009).

Many of these models are based on compelling data derived from a combination of *in vitro* and, to a lesser extent, *in vivo* experiments. In this perspective we will primarily focus on recent reports in human cells and budding yeast that have introduced new players with respect to the control of telomere loop formation. Furthermore, we will speculate on how the regulation of the telomere loop structure needs to be tightly controlled and how it may even provide a relevant target in terms of therapy for diseases associated with deregulated telomere function.

## Mammalian T-loops

T-loops were first reported upon their visualization by electron microscopy on DNA extracted from human and mouse cells (Griffith et al., 1999; Nikitina and Woodcock, 2004). *In vitro*, the telomeric DNA binding protein TRF2 is sufficient to promote t-loop formation (Stansel et al., 2001), although *in vivo* additional factors are likely to be involved (Verdun and Karlseder, 2006). Since TRF1 is able to bend telomeric repeats (Bianchi et al., 1997) and can pair telomeric tracts *in vitro* (Griffith et al., 1998), TRF1 is likely to assist loop formation *in vivo*. It was proposed that TRF1 bends and pairs telomeric duplexes back onto themselves and therefore facilitates strand invasion, catalyzed by TRF2 (Griffith et al., 1999). ssDNA binding protein was detected at the loop junction, arguing that an invasion of the 3′ ssDNA overhang into telomeric double-stranded DNA was occurring and displacing the telomeric duplex (Griffith et al., 1999). Consistently, the presence of a single strand overhang is required for t-loop formation/maintenance *in vitro* (Griffith et al., 1999). Moreover, the recombinogenic proteins RAD51 and RAD52 are present at telomeres during the S/G2 phases of the cell cycle (Verdun and Karlseder, 2006). Strikingly, nuclear extracts from synchronized cells are able to promote *in vitro* telomeric D-loop formation in a RAD51 and RAD52 dependent manner primarily in the S/G2 phases of the cell cycle (Verdun and Karlseder, 2006). The size distribution of t-loops ranges from 3 to 25 kb for HeLa cells and correlates roughly with telomere length. It still remains unclear whether all telomeres carry a t-loop and whether this structure is opened in a cell cycle dependant manner, i.e., to allow the replication fork to pass or to allow telomerase elongation events. One can imagine that a helicase with the ability to resolve D-loops would be an ideal candidate for opening a t-loop when required. Recently, it has been shown that the conserved mouse helicase RTEL1 (regulator of telomere length 1) can undo t-loops *in vitro* (Vannier et al., 2012). In this report, Vannier et al. also reported an accumulation of extra-telomeric circles (t-circles) and increased telomere loss events upon the Cre-mediated removal of RTEL1. They proposed that in the absence of RTEL1, unresolved t-loops accumulate and get processed by the SLX4 nuclease complex when encountered by the DNA replication machinery, thereby liberating a telomere circle and leaving the chromosome end void of telomeric sequence. An independent study reported that the loss of RTEL1 in mouse embryonic stem cells led to gradual telomere shortening, which suggested that RTEL1 loop opening might also be required for telomerase elongation (Uringa et al., 2012). The latter study, however, did not detect increased t-circles upon RTEL1 loss.

**Figure 1:**
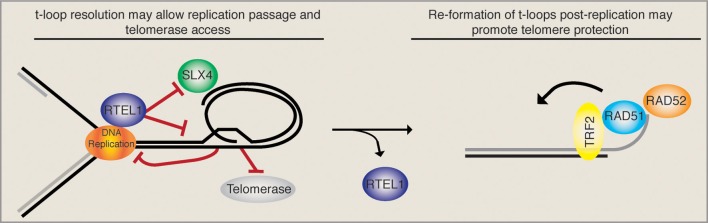
**T-loop regulation may require precise temporal regulation.** The displacement loop likely inhibits the smooth passage of the DNA replication machinery upon telomere replication. It has been suggested that the RTEL1 helicase is able to resolve the t-loop structure and allow replication fork passage while at the same time providing telomerase access to the 3′ ss overhang. In the absence of RTEL1 the replication machinery may encounter the t-loop junction, resulting in fork stalling and the subsequent SLX4-mediated excision of a t-circle, which leads to rapid telomere loss. In this situation (RTEL absent) the loss is compounded by the inability of telomerase to act. Upon the completion of telomere replication the loop must be re-established. Therefore, a complete switch from t-loop resolving to t-loop promoting activities must take place. As the HDR factors RAD51 and RAD52, which are necessary for t-loop formation, get loaded onto telomeres, we suggest that RTEL1 must get actively removed. Together these activities would sequentially promote telomere replication, telomerase elongation, and the re-establishment of a protective loop structure.

Taken together, these data imply that a very intricate temporal regulation must take place upon telomere replication. On the one hand, the t-loop must be opened (possibly by the RTEL1 helicase) upon passage of the replication fork in order to avoid replication collapse and SLX4-mediated generation of t-circles/telomere loss. Loop opening also liberates the 3′ ss overhang that may get extended by telomerase. On the other hand, upon the completion of telomeric replication, the HDR machinery should gain access to the telomere and promote t-loop formation, presumably to maintain telomere protection (see Figure 1). The balance between t-loop resolving and t-loop forming activities must be tightly regulated and presumably depends on rapid and reversible post-translational modifications of the proteins involved. RTEL1, for example, would have to be rapidly removed from telomeres following replication in order to ensure that t-loops can be re-established and are not constitutively unwound. Consistently, it was suggested that RTEL1 is only able to transiently associate with telomeres, however, the mode of regulation remains unknown (Uringa et al., 2012).

## Yeast fold-back structure

For optimal visualization, the TTAGGG repeats of mammalian telomeres were cross-linked by psoralen to prevent branch migrating enzymes from undoing the structures. Looking at telomere fold-backs in *S. cerevisiae* (*Sc*) is complicated by the fact that the yeast telomere repeat sequence lacks a TA step and therefore crosslinking by psoralen before electron microscopy is not possible (de Lange, 2004). Furthermore, yeast telomeres are significantly smaller in size than mammalian telomeres and therefore more difficult to both isolate and visualize. It has been speculated that budding yeast may not have a true telomeric loop due to the absence of TRF1/2-like telomeric proteins, which seem to play a critical role in human t-loop establishment (Tomaska et al., 2004). Furthermore, due to the small size of yeast telomeres, there may be physical restraints on looping back into the telomeric region, which on average is a mere 400 bp in comparison with the approximately 10 kbp telomeres of human cells. Despite these “shortcomings” there is increasing evidence to suggest that the telomeres of budding yeast do indeed loop back, although the molecules involved and the mechanistic details may differ.

Although the TRF1 and TRF2 proteins are absent in budding yeast, compensation has been achieved by uniting the TRF1, TRF2, and RAP1 functions into the single Rap1 protein. Indeed, unlike its human counterpart, budding yeast Rap1 binds telomeric DNA directly via its Myb domain. Furthermore, Rap1, like TRF1, has the ability to bend and reshape DNA molecules (Gilson et al., 1993). Finally, Rap1 is also essential to prevent telomere dysfunction resulting from exonuclease-mediated resection (Vodenicharov et al., 2010), chromosome end fusions (Pardo and Marcand, 2005) as well as rapid telomere deletions (Li and Lustig, 1996). Although yeast Rap1 directly binds only 300 bp of telomeric DNA at the end of the chromosome, it was found in chromatin immunoprecipitation (ChIP) experiments to be associated with subtelomeric regions up to 2 kb away from the telomere, despite the fact that chromatin was sheared to 500 bp fragments or less (Strahl-Bolsinger et al., 1997; Poschke et al., 2012). These experiments suggest that yeast telomeres fold back onto themselves and into the subtelomeric region. A clever genetic trick with a transcriptional readout was employed by the Ptashne lab to further demonstrate a fold-back structure in budding yeast (de Bruin et al., 2001). They modified a telomere so that the *URA3* gene was followed at its 3′ end by the *GAL4 UAS* (upstream activating sequence), which was in turn followed by the telomere (de Bruin et al., 2000, 2001). They demonstrated that in the presence of galactose *URA3* transcription was induced, suggesting that the *UAS* was folding back and activating the natural *URA3* promoter. They confirmed this physical interaction by ChIP and went on to show that the transcriptional activation of *URA3* by a downstream *UAS* was specific to its telomeric localization, where looping back was favored. Interestingly, the fold-back structure at telomeres required the Sir2 lysine deacetylase (KDAC) complex. Subsequently, it has been observed that Cdc13, which binds to the telomeric 3′ overhang in yeast, can also precipitate subtelomeric DNA up to 1.5 kb away from the end despite extensive chromatin shearing. Ku70 has also been implicated in the establishment of telomeric loops in budding yeast (Pryde and Louis, 1999). Taken together, these experiments strongly suggest that yeast telomeres fold back into a loop-like structure.

Despite the increasing body of evidence demonstrating telomere fold-backs in yeast, it remains unclear as to whether the fold-back structure at budding yeast telomeres is equivalent to mammalian t-loops. Firstly, all of the above mentioned experiments suggest that yeast telomeres fold-back into the subtelomere, in contrast to mammalian t-loops that are exclusively comprised of telomere repeat containing DNA. The subtelomeric fold-back may be a requirement due to the shorter yeast telomeres combined with a minimal length requirement for loop formation. It should be noted that yeast subtelomeres contain multiple telomeric repeat sequences throughout their entirety, therefore the 3′ overhang would, in theory, have an ample supply of homologous sequences to basepair with. However, the dependency on the 3′ overhang and whether a displacement loop even exists at yeast telomeres has not been addressed. Our preliminary data suggest that in contrast to human t-loops, *RAD52, RAD51*, and *RAD50* appear to be dispensable for fold-back formation in yeast (Sarah Luke-Glaser and Brian Luke, unpublished data). Due to these striking differences, we refrain from referring to the yeast fold-back at telomeres as a t-loop. Although there are mechanistic differences related to loop establishment between yeast and man, the loop may indeed have similar protective properties for chromosome ends.

**Figure 2:**
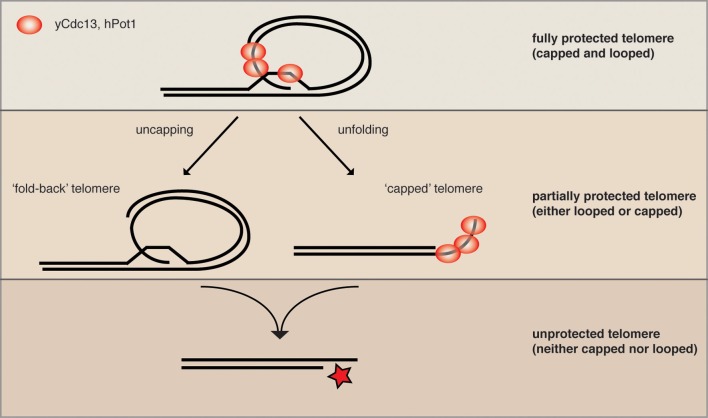
**Parallel pathways may promote end protection.** We speculate that a fully protected telomere (top) exists when the telomere is folded back and the 3′ ssDNA overhang is engaged in a D-loop (not yet shown for yeast). The remaining ss telomeric DNA would then be coated by the organism-specific ss telomeric binding proteins (as depicted here for human and *S. cerevisiae*) to prevent excessive resection and checkpoint activation (capping). When either looping or capping is compromised, the chromosome ends become more vulnerable to resecting nucleases (middle), while some protection is maintained. The simultaneous loss of looping and capping renders telomeres completely open to resection and eventually DNA repair activities, which can lead to chromosome fusions and genome instability.

The yeast helicase Mph1 was proposed to be the functional homolog of mouse RTEL1 as it has the same enzymatic properties *in vitro* (Uringa et al., 2011). Interestingly, overexpressed Mph1 promotes rapid cellular senescence in yeast mutants lacking telomerase activity (Luke-Glaser and Luke, 2012). As Mph1 can disassemble D-loops, the proposed intermediate of a t-loop, *in vitro*, we proposed that Mph1 overexpression may be opening the fold-back structure and exposing telomere ends to nucleolytic attack. Consistently, in telomerase mutants Mph1 overexpression led to increased ssDNA at the telomere and was highly toxic in mutants that were compromised for telomere capping. Overexpression of Mph1 in wild type cells did not have any obvious telomere dysfunction phenotypes. Together, these data would be in line with a model whereby Mph1 would undo a fold-back structure at the telomere, but that telomeres only become completely dysfunctional once they also lack capping function (Figure 2). We propose that telomeric binding proteins such as the CST and KU-complex are the first line of defense and that a higher order structure such as a fold-back acts in parallel to keep telomeres in a protected state.

In order to gain a more global perspective of the genetic requirements for telomere looping, Poschke et al. (2012) used the above-described *URA3* looping reporter and screened the collection of viable yeast deletions for mutants that were unable to induce *URA3* transcription. This approach yielded 112 candidate genes that were defective for telomere looping. Many of the candidates were further confirmed to be looping defective with Rap1 and Cdc13 ChIP assays, as described above. This experiment revealed that the Rap1 associated factors Rif1 and Rif2 were both looping defective; furthermore, the telomere associated KDACs Rpd3, Hda1, and Sir2 were also found to be defective for loop formation. Intriguingly, the mutants on their own had only mild telomere dysfunction phenotypes, which were synergistically exacerbated upon either inhibition of telomerase or inactivation of telomere capping proteins. This is consistent with what was reported when Mph1 was overexpressed and supports the notion that protein-mediated telomere capping and telomere looping may promote end protection in parallel pathways (Figure 2). In addition to the KDAC complexes that were found in the yeast telomere looping screen, there were a surprising number of genes implicated in diverse biological functions ranging from chromatin remodeling to metabolic signaling to translation regulation. Indeed, it is likely that the effects on telomere looping in the majority of these mutants are indirect, however, this range of biological pathways that converge on telomere structure opens the door to many possibilities in terms of manipulating the loop structure (see below). It will be interesting to determine how many of these pathways also affect telomere function in vertebrates.

## Future perspectives—loop control

Targeting telomeres and telomerase for therapeutic purposes is by no means a novel concept. Since all cancer cells rely on continuous telomere maintenance (through either telomerase activation or HDR), it has been suggested that the inhibition of telomere maintenance may cripple their “immortal” properties and drive them into cellular senescence. As cancer cells are highly proliferative and, in general, have shorter telomeres than non-oncogenic cells, it was postulated that such therapy would preferentially target tumor cells. The drawbacks associated with the inhibition of telomere maintenance are: (1) The telomeres may not shorten fast enough in the tumor environment to have an effect on cell viability and (2) the inhibition of telomerase may eventually select for cells that maintain telomeres through HDR (ALT cells). Indeed, a recent study using a mouse tumor model has demonstrated that telomerase inhibition does initially lead to tumor shrinkage, however, this is followed by a relapse whereby the tumor re-grows and in a more aggressive manner (Hu et al., 2012). Upon examining the telomere status in these “relapsed tumors,” it was determined that they had engaged the ALT pathway of telomere maintenance. Therefore, using telomerase inhibitors for cancer therapy may be more effective when combined with a means to increase the rate of telomere loss/resection. This combined therapy would have the advantage of eliminating the “lag time” associated with telomere shortening and furthermore may decrease the window of opportunity for ALT to develop.

Studies in yeast have been able to establish strong correlations between loss of the telomere fold-back structure and increased rates of telomere loss/resection (Poschke et al., 2012) (see above). This suggests that inhibition of the t-loop in combination with telomerase inhibition may result in accelerated telomere loss/resection and thereby promote the rate of senescence in highly proliferative cells where telomere maintenance is essential. Such an approach may also prevent the re-occurrence of ALT cells by: (1) Giving ALT less time to initiate and (2) reducing the amount of t-circles, which have been proposed to arise via t-loop resolution and may act as substrates for HDR mediated telomere elongation. The above-described screen for looping defective yeast mutants may provide a starting block for candidate pathways/molecules that could be either inhibited or activated to inhibit telomere looping. KDACs, for example, emerge as prime candidates due to their high degree of conservation and the availability of small molecule KDAC inhibitors (Khan and La Thangue, 2012). In yeast it has been shown that loss of Rpd3 function (mammalian KDAC1) leads to a looping defect and subsequently to rapid onset of senescence in the absence of telomerase. It was also observed that these mutants were delayed in forming survivors (the yeast ALT equivalent) (Dees and Luke, unpublished data). Of course it remains to be tested whether or not the regulators of telomere looping in yeast are also responsible for modulating the t-loop structure in mammals.

Both RTEL1 and yeast Mph1 have been implicated in telomere function, potentially through the regulation of loop structures. Understanding the regulation of these factors at telomeres may also provide means to manipulate t-loops in conjunction with telomerase inhibition. Whereas the inactivation of RTEL1 leads to accelerated telomere loss due to the inability to resolve t-loops, the over-expression of Mph1 may lead to telomere dysfunction through constitutive loop opening. Indeed, both the hyper- and hypo-activation of RTEL1/Mph1 could be combined with loss of telomerase to promote senescence.

In summary, as we learn more about how telomere structure is established and maintained, we increase the possibilities available for telomere-based therapies. Such therapies could be particularly beneficial to limit the spread of cancer cells where telomere length maintenance is required for continued proliferation.

### Conflict of interest statement

The authors declare that the research was conducted in the absence of any commercial or financial relationships that could be construed as a potential conflict of interest.
